# Validation of the Malay Version of the Decisional Balance Inventory (MDBI) among secondary school-going adolescents

**DOI:** 10.18332/tid/152409

**Published:** 2022-09-19

**Authors:** Kuang Hock Lim, Yoon Ling Cheong, Hui Li Lim, Chee Cheong Kee, Sumarni Mohd Ghazali, Pei Pei Heng, Chien Huey Teh, Mohd Hazilas Mat Hashim, Yong Kang Cheah, Jia Hui Lim

**Affiliations:** 1 Institute for Medical Research, National Institutes of Health, Kuala Lumpur, Malaysia; 2 Clinical Research Centre, Hospital Sultan Ismail, Johor Bahru, Malaysia; 3 Department of Biostatistics and Data Repository, National Institutes of Health, Shah Alam, Malaysia; 4 School of Economics, Finance and Banking, Universiti Utara Malaysia, Sintok, Malaysia; 5 Pharmacy Department, Hospital Putrajaya, Putrajaya, Malaysia

**Keywords:** validation, Malay version, decision balance inventory, school-going adolescents

## Abstract

**INTRODUCTION:**

The Decisional Balance Inventory is a tool used to measure propensity to maintain or change a habit which takes into consideration the perceived advantages and disadvantages. This study aimed to establish the validity and reliability of a Malay language version of the DBI for assessing inclination for change in smoking behavior among secondary school-going adolescents in Malaysia.

**METHODS:**

We administered the MDBI to 669 secondary school students selected through multistage sampling. The sample consisted of 60.1% male (n=398) and 39.9% (n=264) female students, more than two-thirds (69.9%, n=463) of which were from rural areas. The majority of the respondents were aged 13–14 years [13 years, 36.4% (n=241), 14 years, 40.0% (n=265), 16 years, 23.6% (n=156)]. The construct validity of the MDBI was assessed using explanatory (EFA) and confirmatory factor analysis (CFA), and the reliability of the MDBI via Cronbach’s alpha.

**RESULTS:**

EFA and parallel analysis extracted three factors in the MDBI that accounted for 65.4% of the observed variance, and this was supported by CFA. Internal consistency of the three factors ranged from 0.734 to 0.867, indicating acceptable reliability.

**CONCLUSIONS:**

The MDBI has good psychometric properties and is suitable for measuring smoking intention among Malaysian secondary school-going adolescents. However, it should continue to be tested to expand its usefulness and applicability among adolescents in other sociodemographic settings.

## INTRODUCTION

Smoking-related diseases have been the primary causes of premature death and disability in Malaysia for the last three decades^1,2^. Annually, an estimated 20000 deaths are from smoking-related diseases^2^, with approximately a third of the burden of diseases being related to smoking^1^. This rate is expected to increase if the smoking prevalence among Malaysian adults persists^2^.

It is known that smoking is a learned behavior that usually begins during adolescence^3-5^. The National Health and Morbidity Survey 2015 in Malaysia found nearly 70% of smokers began smoking before the age of 18 years^2^. The earlier adolescents start smoking, the more likely they will become smokers in adulthood^6^. Earlier initiation of smoking is also linked to higher risk of smoking-related diseases, such as cancer and cardiovascular diseases^6,7^. Nevertheless, should they quit smoking, they can gradually regain their health^8^. However, relapse of nicotine addiction after stopping makes quitting difficult^9^. Usually, long-term smokers are not easily persuaded by the benefits of quitting smoking, especially when they have yet to be afflicted by smoking-related diseases^10,11^. Thus, reducing the incidence of smoking initiation and increasing smoking cessation among youths is the only way to significantly reduce the prevalence of young smokers and address the health problems of smoking among the Malaysian population^2^.

Behavioral modification models are frequently used successfully to reduce smoking initiation and improve smoking cessation among teenagers. One such model, the Transtheoretical Model (TTM)^12,13^ assesses a person’s willingness to engage in a new and better behavior. It also includes methods for guiding the individual through the process of change. The TTM consists of five essential constructs: stages of change, the process of change, decisional balance, self-efficacy, and temptation, that describe the willingness to change and its advancement via a series of phases^12,13^.

The struggle model, a fundamental process of making a decision connected with specific health behaviors^12,13^, is reflected in the decisional balance construct. The perceived benefits (pros) and perceived barriers (cons) associated with smoking behavior are the decisional balance. Perceived benefits of smoking include helping in coping with stress and irritation and being a pleasurable activity. Therefore, it increases the attraction of smoking, despite the perceived barriers, including its health risks and being an irritation to others. Many studies have found that when a person progresses through the stages of behavioral change, the perceived benefits grow and the barriers decrease. The expectancy theory, which contends that a person’s relative course of action is influenced by the level of his expectation of rewards or failures, is also the foundation of the DBI^14^. The more critical the information considered before making a decision, the more successful the commitment to that decision and the more steadfast the adherence to that decision^15^. Two studies, one by Velicer et al.^16^ and another by Spencer et al.^17^ demonstrated excellent predictive ability of the DBI. Pallonen et al.^18^ developed a short version of the DBI for adolescents, which consisted of 12 items divided into three categories: smoking cons (six items), social pros (three items), and coping pros (three items). Each of these items is assessed on a 5-point Likert scale, with 1=least important and 5=most significant. The DBI has been validated in many countries, including the United States^19^, Bulgaria^20^, and several Asian nations^21-23^. However, a Malay language version of the DBI has not been developed and validated. Given the high incidence of smoking initiation among adolescents^24^ and prevalence of smoking among adults in Malaysia^25^, there is a pressing need for a suitable questionnaire on smoking behavior changes. This study therefore aims to establish the validity and reliability of this scale among Malaysian adolescents.

## METHODS

**Figure 1:**
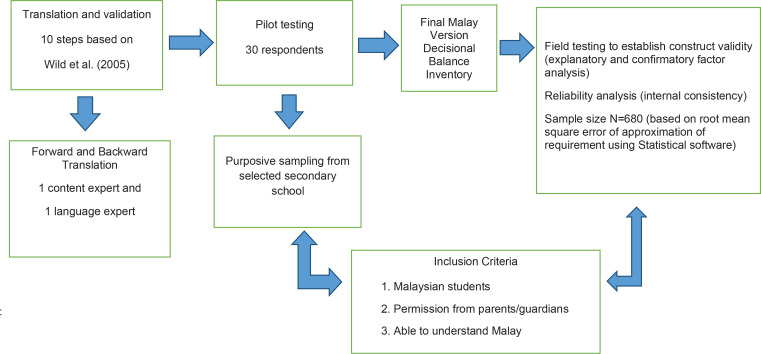
Schematic diagram of methodology to validate MDBI

We used the approach by Wild et al.^26^ to validate the DBI in the Malay language, which consists of translation, followed by assessment of content and face validity, and finally of construct validity (Figure 1).

### Translation

A content expert (public health specialist) and an English language graduate teacher with at least five years of teaching experience translated the DBI into the Malay language, the Malay version was then back translated into English by two other content and language experts. Members of the research team examined both versions, and a harmonized questionnaire was agreed upon by both the translation team and members of the research team. To complete the translation, the team compared the original DBI with the Malay version and some language and cultural adjustments were made. This preliminary Malay version was pilot-tested in 30 male adolescent smokers. The final Malay version of the DBI was produced, and used in this study. Cognitive debriefing was carried out in 30 school-aged adolescents. Respondents were asked to evaluate all items in the DBI from the perspective of difficulty or ambiguity in responding to the Malay version of DBI.

### Content and face validity

We sent the final version of the MDBI to six content experts who were requested to evaluate each item on a Likert type scale of 1–4 in terms of consistency, relevance, representativeness, and clarity (1=not relevant to 4=very relevant, very simple, and very clear). Based on the feedback from the experts, content validity index (CVI), SCVI, and kappa agreement were calculated. The CVI assessed the proportion of items on a scale that attained a rating of 3 or 4 by the experts for each item.

### Construct validity

We established construct validity through a cross-sectional validation study among selected secondary school students in Kota Tinggi, Johor. Two-stage proportionate to size sampling was employed to obtain a sample of students. The first stage was random selection of schools by systematic random sampling, followed by selection of two classrooms from the selected schools using simple random sampling. We invited all students from the classes chosen to participate in the study.

The minimum required sample size was determined for structural equation modelling as follows. The degrees of freedom were calculated based on the number of items in the DBI (12 items), and the number of domains (three domains) as: df = [(number of items - number of domains)×2 - (number of items + number of domains)]/2 = [(12-3)×2 - (12+3)]/2 = 33. Based on the population root mean square error of approximation (RMSEA) of 0.075, null hypothesized RMSEA≤0.05, alpha (Type 1 error) of 0.05, and power of 0.80, the required sample size was 518. The sample size was inflated with an additional 30%, for non-response, to 674.

### Protocol

We employed the active consent approach in this study. Letters containing information about the study (i.e. objective, content of the study, voluntary participation principle, use of the information for research purposes) and consent forms were sent through the school to the selected students’ parents/guardians. Only selected respondents whose parents/guardians consented in writing were admitted into the study. A self-administered paper-and-pencil questionnaire (Supplementary file) was distributed during school hours in the presence of a member of the research team who explained the purpose and procedure of the study. Participants were assured that their feedback was anonymous and confidential and that they could quit the study at any given time. Data collection took approximately 20–30 minutes to complete. The study was granted ethical approval from the Medical Research and Ethical Committee of the Malaysian Ministry of Health and the Malaysian Ministry of Education.

### Data management and analysis

**Figure 2:**
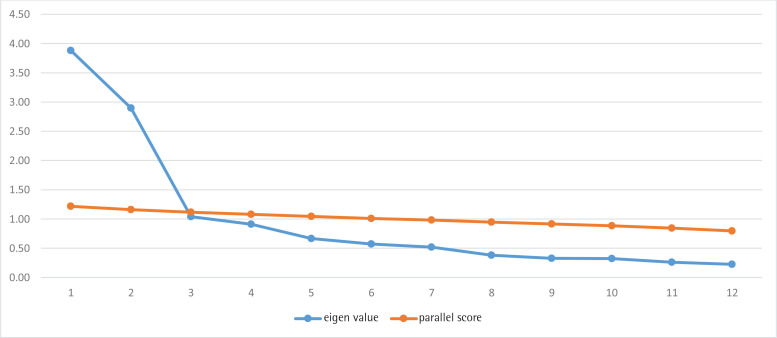
Eigen value and parallel score analysis to determine the number of domains in MDBI

The data were cleaned before any statistical analysis was carried out. Descriptive statistics was used to describe the characteristics of the respondents. We calculated item-level Content Validity Index (I-CVI), scale-level content validity index (SCVI), and kappa statistic for agreement to assess content validity based on the experts’ assessment of the questionnaire. Exploratory factor analysis was performed to determine the construct validity of MDBI. The ideal number of factors was determined using eigenvalue above 1 as criterion. Parallel analysis was used to determine the number of domains in MDBI (Figure 2) using Varimax rotation and factor loadings of ≥0.3 as the criterion for item inclusion. Kaiser Mayer-Olkin and Bartlett’s tests were used to assess adequacy of the data. Determination of the number of domains was by examination of the meeting point of the variance generated by SPSS and FACTOR.

Following this, confirmatory factor analysis (CFA) was performed to validate the structure elucidated by EFA. Model fit was evaluated using multiple fit indices, namely relative chi-squared, goodness of fit index (GFI), comparative fit index (CFI), root mean square error of approximation (RMSEA), non-normed fit index (NNFI), and normed fit index (NFI). Construct validity and discriminant validity in CFA were measured by the average variance and the construct reliability^27^. Reliability of the MDBI was assessed by examining the total correlation and the impact of removing each item. SPSS software was used to run the EFA and reliability analysis, FACTOR freeware (version 12.01.02; Lorenzo-Seva & Ferrando, 2006)^28^ to determine the number of domains that needed to be extracted, and SPSS AMOS software to perform the CFA. All statistical analyses were performed at the 95% significance level.

## RESULTS

Table 1 shows the content validity results for the second session after improvements were made to the questionnaire based on the comments of the content experts. The results showed that five items obtained a score of 3 or 4 from all content experts, while the remaining seven items obtained a score of 3 or 4 from only five of the experts. The CVI coefficients ranged from 0.30 to 1.00, while modified kappa varied from 0.816 to 1.00. The SCVI/UA was 0.4167, and the SCVI/Ave exceeded the set cut-off value of 0.90.

**Table 1:** Content validity index I-CVI, modified kappa, and S-CVI by two approaches of S-CVI/UA and S-CVI/Ave for items of MDBI after the second round of judgment

*Item*	*Relevance rating 3 or 4*	*Relevance rating 1 or 2*	*I-CVI*	*Modified kappa*	*Interpretation*
1	5	1	0.83	0.816	Accepted
2	6	0	1.00	1	Accepted
3	6	0	1.00	1	Accepted
4	5	1	0.83	0.816	Accepted
5	6	0	1.00	1	Accepted
6	5	1	0.833	0.816	Accepted
7	6	0	1.00	1	Accepted
8	5	1	0.83	0.816	Accepted
9	5	1	0.83	0.816	Accepted
10	5	1	0.83	0.816	Accepted
11	6	1	0.83	0.816	Accepted
12	6	0	1.00	1	Accepted

I-CVI: item-level content validity index. S-CVI/Ave = 0.915 (S-CVI/Ave is calculated by taking the sum of the I-CVIs divided by the total number of items). S-CVI/UA = 0.416 (S-CVI/UA is calculated by adding all items with I-CVI equal to 1 divided by the total).

A total of 662 students responded, giving a high response rate of 98.2%. Of the 662 respondents, 3 out of 5 were male (n=389), and almost 70% (n=463) were from rural secondary schools. Most of the respondents were of Malay descent (86.3%, n=571), followed by Chinese (10.6%, n=70), and the remainder were of Indian and other ethnicities. Approximately three-quarters of the respondents were not current smokers (Table 2). Exploratory factor analysis based on eigenvalues above 1 and parallel analysis with FACTOR identified three domains. The total variance explained by the three domains was 65.57%. The first domain consisted of six items (accounting for 30.8% of the total variance), the second domain had three items (17.7%), while the third domain had three items (17.1%) (Table 3).

**Table 2:** Sociodemographic characteristics of the respondent school-going adolescents in Kota Tinggi, Johor, Malaysia

*Characteristics*	*n*	*%*
**Gender**		
Male	398	60.1
Female	264	39.9
**Age** (years)		
13	241	36.4
14	265	40.0
16	156	23.6
**Locality**		
Urban	199	30.1
Rural	463	69.9
**Smoking status**		
Current smoker	157	24.6
Non-smoker	482	75.4

**Table 3:** Exploratory factor analysis of the MDBI among 662 school-going adolescents in Kota Tinggi, Johor, Malaysia

*Number*	*Domain 1 (Cons scale)*	*Domain 2 (Social pro)*	*Domain 3 (Coping pro)*
1	Smoking can affect the health of others		
2	Smoking stinks		
3	Smoking cigarettes is hazardous to people’s health		
4	Cigarette smoking bothers other people		
5	Smoking is a messy habit		
6	Smoking makes teeth yellow		
7		Smoking makes kids get more respect from others	
8		Kids who smoke have more friends	
9		Kids who smoke go out on more dates	
10			Smoking helps people to cope better with frustrations
11			Smoking cigarettes is pleasurable
12			Smoking cigarettes relieves tension
**Variance explained**			
	30.82	17.70	17.05

Kaiser-Meyer-Olkin measures of sampling adequacy 0.815. Bartlett’s test of sphericity, χ^2^=3415.54, df=66, p<0.001.

**Figure 3:**
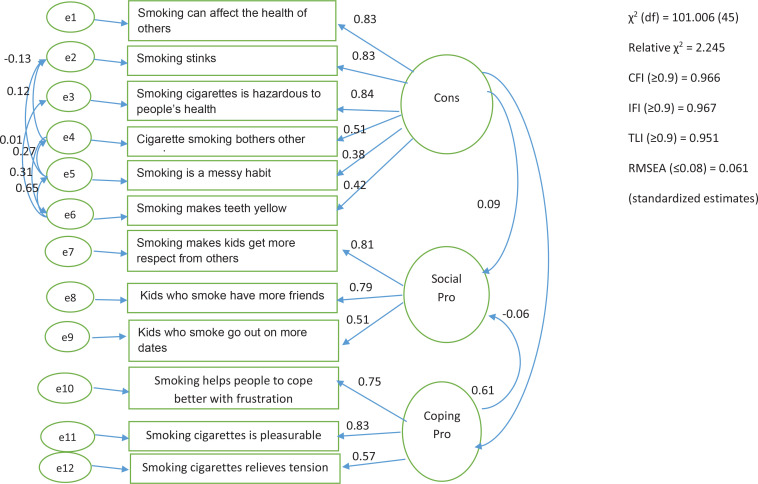
Schematic diagram of MDBI validation methodology

Figure 3 shows results of the CFA, which showed correlation coefficients between 0.51 and 0.81 between the items and the latent social pros domain and a comparable coefficient value in the coping pros domain. In the cons domain, two items had low correlation coefficients, but both were retained in the questionnaire at the suggestion of the content specialists. The RMSEA value was 0.061 (0.08), the relative chi-squared was 2.245 (5.00), while the CFI, ILI, and GFI values exceeded 0.90, indicating a good fit of the model. Table 4 shows that the average variance explained was >0.50 for the coping pros and social pros domains, but only 0.468 for the cons domain.

**Table 4:** Construct reliability and average variance explain of Malay Decision Balance Inventory among 662 school-going adolescents in Kota Tinggi, Johor, Malaysia

*Factor*	*Item*	*Construct reliability*	*Average variance extracted*
**Cons of smoking**	Smoking can affect the health of others	0.827	0.468
	Smoking stinks		
	Smoking cigarettes is hazardous to people’s health		
	Cigarette smoking bothers other people		
	Smoking is a messy habit		
	Smoking makes teeth yellow		
**Social pro**	Smoking makes kids get more respect from others	0.753	0.513
	Kids who smoke have more friends		
	Kids who smoke go out on more dates		
**Coping pro**	Smoking helps people to cope better with frustrations	0.764	0.526
	Smoking cigarettes is pleasurable		
	Smoking cigarettes relieves tension		

## DISCUSSION

This study aimed to assess the psychometric features of the MDBI among secondary school students. The findings suggest that the translated DBI is a suitable and valid questionnaire for assessing smoking versus non-smoking decision-making among Malaysian school-aged teenagers. CVI and kappa for agreement demonstrated the MBDI content’s validity was at an acceptable level. Furthermore, as performed by Pallonen et al.^18^, EFA and parallel analysis showed that MDBI is divided into three domains. In addition, the communalities in each domain are comparable to one another. The first version’s explained variation was greater than the prescribed 50% level. The results of the CFA corroborated the conclusions of EFA and parallel analysis. In addition to the domains’ reliability, which ranged from 0.753 to 0.857, the item-to-subtotal correlation coefficients for the subscales were greater than 0.45, indicating a satisfactory level of reliability.

The results also showed that the questionnaire could distinguish between different domains in the MBDI and measure various aspects of the disadvantages of smoking (e.g. concerns for smoking effects on health). However, the results contradicted the findings of Chen et al.^23^, who found only two domains in the Chinese version of the DBI for Taiwanese adolescents, and Hoeppner et al.^19^, who discovered four factors (two positive and two negative) among African American adolescents. This discrepancy could be due to the different characteristics of the respondents in this study. For example, the study by Hoeppner et al.^19^ involved only females, versus the present research which consists of both males and females in a 3:2 ratio.

The three domains accounted for 65.4% of the variance. This is higher than the 55.4% previously report by Khazaee-Pool et al.^22^ and Velicer et al.^16^. In the Velicer et al.^16^ analysis, the solution of two components (i.e. advantages and disadvantages) accounted for 41% of the observed variance, which is higher than in the study of Pallonen et al.^18^ where three-factor solutions (social benefits, overcoming advantages, and drawbacks) accounted for half of the variance. Hoeppner et al.^19^ found a four-factor solution (two pro factors and two cons factors) that explained 45% of the variance, which was lower than in Chen et al.^23^ (74.5%). This could be explained by more items in the two DBI domains found in the results of the study among adolescents in Taiwan.

CFA analysis of the MDBI revealed a positive link between social and coping pros, with a correlation of 0.61 in this study. This is consistent with other studies using the same instrument. The Plummer et al.^29^ correlation coefficient was virtually the same at 0.59. The poor correlation between social coping and the cons of smoking is also consistent with earlier research.

The Cronbach alpha coefficients for the smoking cons scale, the social pros scale, and the coping pros scale were 0.867, 0.754, and 0.753, respectively (Table 5). These reliability estimates are similar to the coefficients obtained in the study for these same scales, which were 0.80 for smoking, 0.787 for social pros, and 0.832 for coping pros by Khazaee-Pool et al.^22^. These reliability estimates are consistent with the alpha obtained in the Plummer et al.^29^ study for the same scales, in which the alphas for social pros, coping pros and cons were 0.68, 0.79 and 0.86, respectively.

**Table 5:** Reliability analysis for MDBI among 662 school-going adolescents in Kota Tinggi, Johor, Malaysia

*Item*	*Domain/item*	*Mean*	*SD*	*Item-total correlation*	*Cronbach alpha if item is deleted*	*Cronbach alpha*
	**Cons of smoking**					
1	Smoking can affect the health of others	1.61	0.92	0.68	0.84	0.867
5	Smoking stinks	1.51	0.88	0.69	0.84	
6	Smoking cigarettes is hazardous to people’s health	1.57	0.84	0.70	0.84	
9	Cigarette smoking bothers other people	1.75	0.91	0.63	0.85	
11	Smoking is a messy habit	1.94	1.03	0.62	0.85	
12	Smoking makes teeth yellow	1.82	0.98	0.68	0.84	
	**Social pros**					
2	Smoking makes kids get more respect from others	3.45	0.77	0.46	0.80	0.754
8	Kids who smoke have more friends	3.25	0.879	0.66	0.58	
10	Kids who smoke go out on more dates	3.14	0.875	0.65	0.60	
	**Coping pros**					
3	Smoking helps people to cope better with frustrations	2.96	1.01	0.62	0.62	0.753
4	Smoking cigarettes is pleasurable	3.12	0.93	0.67	0.56	
7	Smoking cigarettes relieves tension	3.50	0.79	0.47	0.78	

### Limitations

This study has some limitations. For example, the study included only secondary school students in the Kota Tinggi District. Therefore, the majority of Malay students do not reflect the racial composition of this country, nor can it be generalized to adolescents of different social backgrounds and localities. In addition, smoking status among respondents is self-reported, which may lead to under- or over-reporting of smoking status. Furthermore, differences between Malaysian youth culture and the culture of the population for which the DBI was initially developed could necessitate additional items being added to the measure.

## CONCLUSIONS

Overall, the findings suggest that the Malay version of the DBI is a reliable and valid scale for assessing adolescents’ consideration of whether to smoke or not. The MDBI should be further tested on adolescents in various sociodemographic and geographical settings to confirm its applicability in the general Malaysian adolescent population.

## Data Availability

The data supporting this research are available from the authors on reasonable request.
