# Supplementary material for: Validation of the Malay Version of the Decisional Balance Inventory (MDBI) among secondary school-going adolescents
Source: Tob Induc Dis. 2022 Sep 19;20:79. doi: 10.18332/tid/152409 (PMC9483825; doi:10.18332/tid/152409)
Supplement: Supplementary file 1 [file TID-20-79-s1.pdf]

*Appendix 1*

| Item | Item description | Has the item in the Instrument Consistency | Are the Instrument representative of concept of the topic | Are the items relevance to the concept of the topic | Are the items clarity in term of wording | Comments |
|---|---|---|---|---|---|---|
| 1 | Smoking can affect the health of others | | | | | |
| 2 | Smoking makes kids get more respect from others | | | | | |
| 3 | Smoking helps people to cope better with frustrations | | | | | |
| 4 | Smoking cigarettes is pleasurable | | | | | |
| 5 | Smoking stinks | | | | | |
| 6 | Smoking cigarettes is hazardous to people’s health | | | | | |
| 7 | Smoking cigarettes relieves tension | | | | | |
| 8 | Kids who smoke have more friends | | | | | |
| 9 | Cigarette smoking bothers other people | | | | | |
| 10 | Kids who smoke go out on more dates | | | | | |
| 11 | Smoking is a messy habit | | | | | |

| | | | | | | |
|---|---|---|---|---|---|---|
| 12 | Smoking makes teeth yellow | | | | | |
| 1 - Not relevant 2 - Somewhat relevant 3 - quite relevant 4 - Highly relevant | | | | | | |

© 2022 Lim K.H. et al.
